# Evaluating Hepatitis B Screening During Pregnancy: A Study on Diagnostic Accuracy and Infection Control in Nigeria

**DOI:** 10.1111/jvh.70002

**Published:** 2025-01-20

**Authors:** Chinomso Joel Ukagebu, Jude Oluwapelumi Alao, Favour Oluwadara Bamigboye, Joel Chimezie Ukaegbu, Elijah Kolawole Oladipo

**Affiliations:** ^1^ Adeleke University Ede Osun Nigeria; ^2^ Auckland University of Technology Auckland New Zealand; ^3^ Helix Biogen Institute Ogbomosho Oyo Nigeria

**Keywords:** antenatal care, hepatitis B virus, infection control, Nigeria, seroprevalence

## Abstract

Hepatitis B virus (HBV) remains a critical public health issue in low‐ and middle‐income countries (LMICs), particularly among pregnant women in Nigeria. Routine screening using rapid diagnostic kits is common in antenatal care, yet the accuracy of these tests can vary. This study aimed to determine the seroprevalencwe of HBV among pregnant women who had previously undergone screening using rapid diagnostic kits at Obafemi Awolowo Teaching Hospital, Ilesa, Osun State, Nigeria, to assess the effectiveness of initial screening and identify any missed cases. A cross‐sectional study was conducted, involving 263 pregnant women. Blood samples were tested for HBV markers (HBsAg, HBsAb, and HBcAb) using ELISA. Sociodemographic data and potential risk factors were also analysed. The study found that 7.6% of women were HBsAg positive, indicating active HBV infection, and 49.6% were susceptible to HBV. There was a significant association between higher education levels and HBV seropositivity. Employment status also correlated with HBV prevalence, with self‐employed women showing higher seroprevalence. Additionally, a history of blood transfusions was linked to higher HBV seropositivity. The findings highlight the limitations of rapid diagnostic kits in detecting HBV and underscore the need for enhanced infection prevention and control measures, including confirmatory testing, robust vaccination programmes and safe delivery practices to reduce HBV transmission in high‐burden regions like Nigeria.

## 1 Introduction

Hepatitis B virus (HBV) infection remains a significant global health challenge, particularly in low‐ and middle‐income countries (LMICs), where it contributes substantially to morbidity and mortality. With an estimated 296 million individuals living with chronic HBV infection as of 2023, the burden on healthcare systems is immense, especially in regions with limited resources [1]. The consequences of HBV are severe, leading to approximately 820,000 deaths annually due to liver‐related complications such as cirrhosis and hepatocellular carcinoma [2]. The World Health Organisation (WHO) has identified the elimination of HBV as a public health threat by 2030 as a critical global priority, particularly in high‐prevalence regions like sub‐Saharan Africa [3].

In LMICs, chronic hepatitis B (CHB) represents not only a public health threat but also a significant challenge for infection prevention and control (IPC) within healthcare settings. The virus is primarily transmitted through contact with infected blood and bodily fluids, with vertical transmission (from mother to child during childbirth) and horizontal transmission (from person to person) being the predominant routes in these regions [4, 5, 6]. In healthcare facilities, the risk of HBV transmission is heightened by factors such as inadequate screening practices, limited access to vaccination and insufficient infection control protocols.

Nigeria, the most populous country in Africa, faces substantial challenges in controlling HBV transmission, both in the community and within healthcare settings. The national prevalence of HBV is approximately 12.2%, with even higher rates observed among pregnant women, at 14.1% [7, 8]. In regions such as Bayara, Bauchi State, the prevalence among pregnant women can be as high as 17.2% [9]. These high prevalence rates highlight the critical need for effective infection prevention strategies in antenatal care settings, where the risk of vertical transmission is particularly high.

Routine hepatitis B screening using rapid diagnostic kits is commonly employed in antenatal care settings in Nigeria. However, the accuracy and sensitivity of these rapid tests can vary, potentially leading to underdiagnosis or false‐negative results, which could hinder effective HBV management and infection prevention efforts. Given these concerns, it is important to assess the true seroprevalence of HBV among pregnant women who have already undergone initial screening.

This study aimed to determine the seroprevalence of HBV among pregnant women who have already been screened using rapid diagnostic kits at Obafemi Awolowo Teaching Hospital, Ilesa, Osun State, Nigeria. This approach is justified by the need to verify the accuracy of initial screening results and to identify any cases of HBV that may have been missed by rapid testing. Accurate determination of seroprevalence is crucial for informing effective IPC strategies, particularly in settings where vertical transmission of HBV remains a significant risk.

By providing updated, region‐specific data, this research informs the development of targeted infection control strategies essential for the effective management and prevention of HBV transmission within healthcare facilities. Addressing the specific challenges faced in LMICs like Nigeria, this study contributes to the broader goal of HBV elimination and the improvement of maternal and child health outcomes in high‐burden regions.

## 2 Materials and Methods

### 2.1 Study Design

This cross‐sectional study was conducted at Obafemi Awolowo Teaching Hospital, Ilesha, Osun State, Southwest, Nigeria.

### 2.2 Ethical Approval

All procedures performed in studies involving human participants were in accordance with the ethical standards of the institutional and/or national research committee, as well as the 1964 Helsinki Declaration and its later amendments or comparable ethical standards. The study received approval from the Osun State Ministry of Health (OSHREC/569 T/309) and the Adeleke University Ethical Committee (AUERC/FOS/MICROBIOLOGY/2022/01). All participants aged 18 years and above provided written informed consent before enrolment. For participants under 18 years, written informed consent was obtained from their parent or legal guardian.

### 2.3 Inclusion and Exclusion Criteria

#### 2.3.1 Inclusion Criteria

- Pregnant women: The study included only pregnant women attending antenatal care at Obafemi Awolowo Teaching Hospital, Ilesa, Osun State, Nigeria.
- Age range: Participants were between the ages of 15 and 50 years
- HBV screening status: Participants must have tested negative for hepatitis B using rapid diagnostic testing during antenatal screening.
- Previous HBV screening: Participants must have previously undergone routine hepatitis B screening using rapid diagnostic kits as part of their antenatal care.
- Residency: Participants had to have been residents of Osun State for at least 1 year to ensure they were representative of the local population.
- Willingness to participate: Participants were required to complete the self‐administered questionnaire and provide necessary demographic and health information.
- No recent HBV vaccination: Participants must not have received an HBV vaccination within the last 12 months to prevent confounding the seroprevalence results, as recent vaccination could elevate HB antibodies. This criterion ensures that detected antibodies are due to prior exposure rather than recent immunisation, providing a more accurate measure of natural seroprevalence.

#### 2.3.2 Exclusion Criteria

- Severe health conditions: Women with severe concurrent health conditions that could interfere with participation or affect the study results, such as severe anaemia, AIDS or autoimmune diseases, were excluded.
- Previous participation: Women who had previously participated in a similar HBV study within the past year were excluded to prevent duplicate data.
- Occupational bias: Medical and religious birth attendants at the selected study sites were excluded to avoid potential bias related to occupational exposure to HBV.

### 2.4 Demographic Data Collection

From December 2022 to June 2023, participants were enrolled for this study. Data were collected on demographic variables [10] (age, education, and marital status) and risk assessment variables (HBV transmission routes, prevention methods, age at first sex, casual and regular sexual partners in the past 3 months, multiple sexual partners and history of sexually transmitted infections in the last 3 months) using an anonymous self‐administered questionnaire. The questionnaire also examined knowledge about HBV infection, previous HBV testing, treatment history, HBV as a cause of liver cancer, prevention of HBV through vaccination and transmission via sexual and blood routes.

### 2.5 Blood Sample Collection

A volume of 5 mL of blood was collected from the study participants and transferred into labelled microtiter tubes containing EDTA. The samples were then centrifuged at 3000 rpm for 10 min to separate the serum. The serum was carefully decanted into new labelled tubes and stored at −80°C until analysis.

### 2.6 Serology Test

Samples were screened for hepatitis B surface antigen (HBsAg), hepatitis B surface antibody (HBsAb) and hepatitis B core antibody (HBcAb) using third‐generation enzyme‐linked immunosorbent assay (ELISA) kits (Melsin Medical Co. Limited, China) in accordance with the manufacturer's recommendations. The selection of these markers—HBsAg, HBsAb and HBcAb—was essential for a comprehensive understanding of HBV infection status among the pregnant women studied. Specifically, HBsAg indicates active HBV infection, HBsAb reflects immunity either through past infection or vaccination and HBcAb reveals past or ongoing infection, providing insights into infection history.

### 2.7 Data Analysis

Descriptive analysis was conducted according to the study objectives. Data were recorded in MS Excel and analysed using Jupyter Notebook (7.0.8). Distribution was established for each participant's samples. Quantitative variables (marital status, occupation and age) were expressed, and qualitative variables were reported as frequencies (percentage). Chi‐square tests were performed to assess the significance of distributions among variables. Significant differences and associations were evaluated at a 95% confidence level (*p* < 0.05). Findings were presented in tables and graphs. Chi‐square tests were used for categorical data to estimate how related the distribution of a categorical variable matches an expected distribution or to assess whether two categorical variables are independent. Logistics regression analysis determined the correlation between demographic factors and seropositivity to HBV markers. A *p* value of < 0.05 was considered statistically significant.

## 3 Results

A total of 263 pregnant women were recruited for this study at Obafemi Awolowo Teaching Hospital, Osun State, Nigeria. The sociodemographic characteristics of these participants are presented in Table 1. Of the participants, 144 (60.5%) were aged between 21 and 30 years, 158 (67.2%) were self‐employed and 156 (65.8%) were graduates of tertiary institutions. Regarding the trimester distribution, 56 (23.6%) were in their first trimester, 128 (54.0%) were in their second trimester and 53 (22.4%) were in their third trimester.

**TABLE 1:** Sociodemographic characteristics of pregnant women.

Variable	Category	Frequency	(%)
Age range (years)	16–20	5	2.1
	21–30	144	60.5
	31–40	84	35.3
	Above 40	5	2.1
Employment status	Unemployed	25	10.6
	Self‐employed	158	67.2
	Employed	52	22.1
Educational level	Primary	13	5.5
	Secondary	67	28.3
	Tertiary	156	65.8
	Not educated	1	
Trimester	1st trimester	56	23.6
	2nd trimester	128	54.0
	3rd trimester	53	22.4

The seroprevalence of HBV markers among the study participants is detailed in Table 2. The data indicated that 124 (49.6%) of the samples were susceptible to HBV, 15 (5.7%) were immune due to natural infection, 6 (2.3%) were vaccinated, 20 (7.6%) were infected and 85 (32.3%) had indeterminate results. An ‘indeterminate’ result, as defined in the test manual, indicates an ambiguous serological outcome that does not clearly confirm or rule out HBV infection but differs from an invalid test result.

**TABLE 2:** Seroprevalence of HBV markers among study participants.

Interpretation	HBsAg	HBsAb	HBcAb	Frequency (%)
Susceptible	Negative	Negative	Negative	124 (49.6%)
Immune due to natural infection	Negative	Positive	Positive	15 (6.0%)
Vaccinated	Negative	Positive	Negative	6 (2.4%)
Infected	Positive	Negative	Positive	20 (8.0%)
Indeterminate	Negative	Negative	Positive	85 (34.0%)

Among the examined samples, 21 (8.0%) were positive for HBsAb while 242 (92.0%) were negative. Additionally, 20 (7.6%) were positive for HBsAg, while 243 (92.3%) were negative. Furthermore, 122 (46.4%) were positive for HBcAb, while 141 (53.6%) were negative. Thus, the prevalence of HBsAb was 8.0%, HBsAg was 7.6% and HBcAb was 46.4%.

**FIGURE 1:**
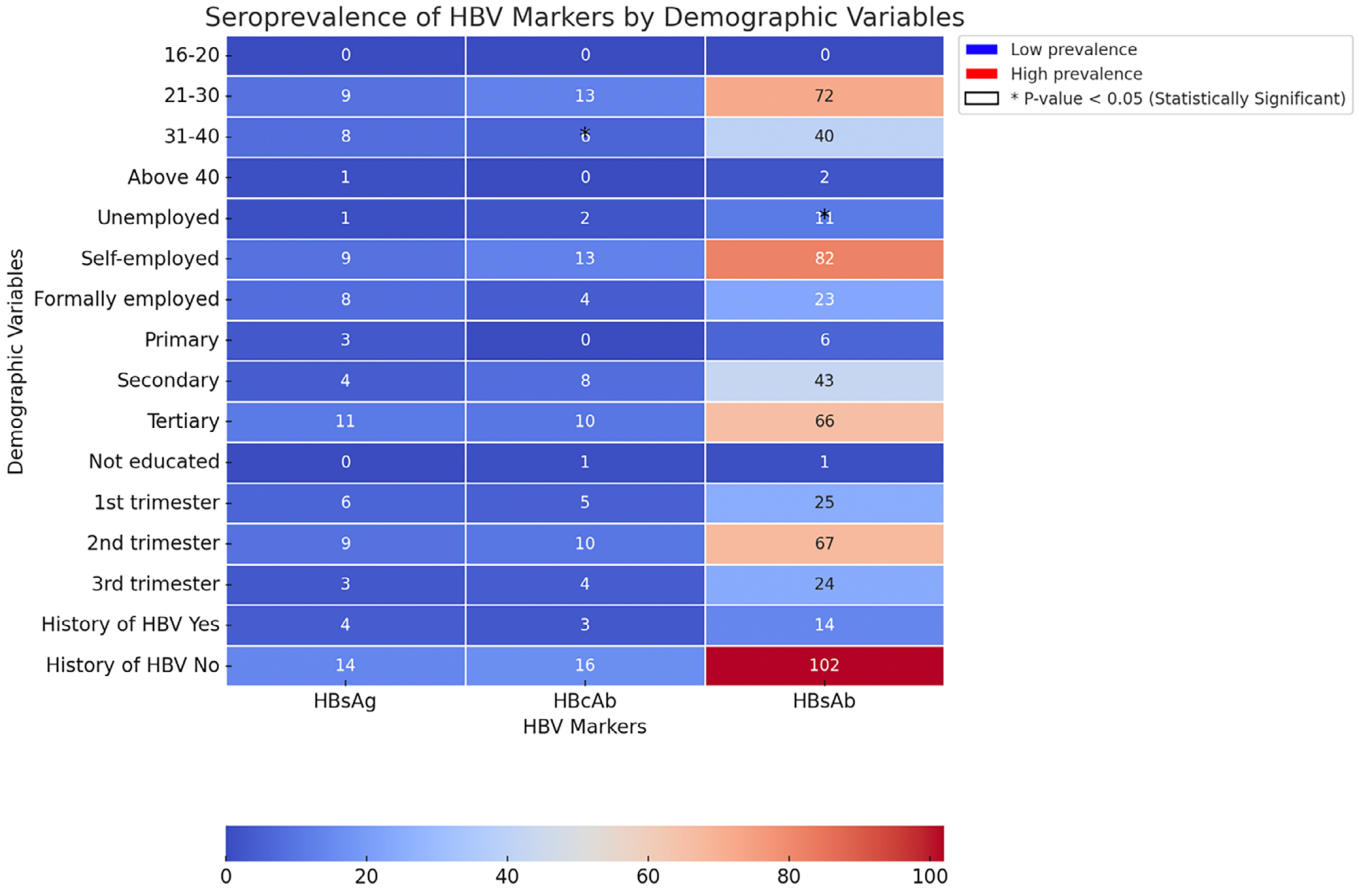
Seroprevalence of variables against HBV markers.

The educational level was statistically significant (*p* = 0.001) in terms of seropositivity, with women having tertiary education showing the highest seropositivity (Figure 1). There was no significant relationship between age and infection (*р* = 0.851). The prevalence of HBV was highest among women aged 21–30 years, followed by those aged 31–40 years.

The study also found that employment status was associated with seropositivity, with self‐employed women having the highest positive results. HBsAb prevalence was highest among pregnant women in their tertiary stages of education.

The study showed no significant correlation between trimester, past use of blood transfusions, past use of surgery, past use of IV drugs and knowledge of HBV (*p* > 0.05). However, 14.2% of seropositive women had received blood transfusions in the past compared to 8.4% who had not.

Higher HBV seroprevalence was seen among pregnant women with just one past sexual partner, although this was not statistically significant (*р* = 0.059).

## 4 Discussion

This study aimed to determine the seroprevalence of HBV among pregnant women attending antenatal care at Obafemi Awolowo Teaching Hospital in Ilesa, Osun State, Nigeria, focusing specifically on women who had already undergone routine screening using rapid diagnostic kits. The findings reveal a significant burden of HBV infection, with a notable proportion of women either currently infected or at risk. These results have critical implications for IPC practices in antenatal care settings, particularly in LMICs like Nigeria.

The high seroprevalence of HBV among the study participants, despite prior screening with rapid diagnostic kits, highlights limitations in the sensitivity of these kits, which may fail to detect all cases of HBV. Even highly sensitive rapid tests can yield false‐negative results, as they are operator dependent. Inaccuracies can arise if the test procedure deviates from the manual—such as using nonstandard pipettes, incorrect diluents or incorrect blood volumes—which may negatively impact the results. This underscores the need for confirmatory testing using more sensitive methods like ELISA in antenatal care protocols. Identifying HBV‐positive mothers early allows timely interventions, including administering hepatitis B immunoglobulin (HBIG) and the first HBV vaccine dose to newborns, proven strategies for preventing vertical transmission.

In addition to enhanced screening, implementing systematic infection control practices is essential for preventing HBV transmission in healthcare settings. Healthcare workers must consistently adhere to biosafety protocols during labour and delivery—such as using personal protective equipment (PPE) and safely handling blood and bodily fluids—regardless of a known infection risk. Postnatal care should also include follow‐up vaccinations for newborns to ensure long‐term immunity against HBV.

The study's findings highlight the importance of implementing effective HBV prevention protocols in healthcare facilities, especially in LMICs where resources are often limited. The significant proportion of women found to be susceptible to HBV, despite prior screening, suggests a gap in vaccination coverage that needs urgent attention. Strengthening immunisation efforts, particularly targeting pregnant women and their infants, can significantly reduce HBV incidence and improve public health outcomes.

Moreover, the study identified a correlation between previous blood transfusions and higher HBV seropositivity, emphasising the need for stringent blood transfusion practices. Healthcare facilities must ensure that all blood products are rigorously screened for HBV and other bloodborne pathogens before transfusion. Adherence to standard precautions, such as the use of sterile equipment and safe injection practices, is critical to preventing nosocomial transmission of HBV in clinical settings.

The results of this study are consistent with a study which identified a history of tonsillectomy, tattoos, multiple sexual partners and contact with jaundiced patients as significant risk factors for HBsAg positivity [11]. However, the HBsAg prevalence in this study (7.6%) is notably lower than the 17.2% reported by Ndako et al. (2012) in Bayara, Bauchi State, which may be attributed to regional differences in HBV transmission dynamics or variations in study populations.

When compared to other LMICs, the HBV seroprevalence rates in this study are similar to those reported in southern Ethiopia, where [12] found high HBV seroprevalence among pregnant women, particularly those with lower educational levels. Similarly, in Northeast Egypt [13], reported prevalence rates of 18.3% for HBcAg, 5.0% for HBsAg and 30.7% for HBsAb. The lower HBsAg prevalence in Egypt might be due to more effective vaccination programmes, underscoring the importance of robust immunisation efforts in reducing the HBV burden.

In contrast, the HBV prevalence in this study is significantly higher than in developed countries, where rigorous vaccination programmes and better healthcare infrastructure have led to much lower incidence rates. For example, in the United States, the prevalence of chronic HBV infection among pregnant women is < 1% [14], highlighting the critical role of comprehensive public health strategies in controlling HBV.

Interestingly, this study found a significant association between educational level and HBV seropositivity, with higher seropositivity observed among women with tertiary education. This contrasts with findings from southern Ethiopia, where illiterate women had higher seropositivity rates [12]. The higher seropositivity among educated women in this study may reflect better access to healthcare services, leading to more frequent testing and diagnosis. Employment status also emerged as a significant factor, with self‐employed women showing the highest seroprevalence. This finding could be linked to socioeconomic factors influencing access to health information and services, suggesting the need for targeted public health interventions tailored to different occupational groups.

The study found no significant correlation between the trimester of pregnancy and HBV markers, indicating that the risk of HBV infection does not vary significantly across different stages of pregnancy. This finding aligns with other studies, reinforcing the importance of continuous HBV screening throughout pregnancy to ensure early detection and management.

A notable observation was that 14.2% of seropositive women had a history of blood transfusions compared to 8.4% who did not. This aligns with existing literature indicating that blood transfusion is a significant risk factor for HBV transmission, further underscoring the need for stringent screening protocols in blood donation practices to mitigate this risk.

This study has several limitations that should be considered when interpreting the findings. Firstly, the lack of genetic analysis, such as genotyping of HBV strains, means that potential variations in the virus that could influence its transmission or response to treatment were not examined. Secondly, the study's cross‐sectional design limits the ability to establish causal relationships between the observed factors and HBV seropositivity. Additionally, the reliance on self‐reported data regarding past blood transfusions, sexual history and healthcare practices may have introduced recall bias or incomplete reporting. The sample size, while relatively large, was limited to a single teaching hospital, which may not fully represent the broader population of pregnant women in Nigeria or other LMICs. Lastly, while the study explored several sociodemographic and behavioural factors, it did not account for other potential risk factors such as coinfections or the exact nature of healthcare access, which could further influence HBV seroprevalence in the studied population.

## 5 Conclusion

This study reveals a significant burden of HBV among pregnant women in Southwestern Nigeria, despite prior screening with rapid diagnostic kits. The findings underscore the need for enhanced IPC measures, including more accurate screening methods, robust vaccination programmes and strict adherence to safe delivery practices. Addressing these gaps is crucial for reducing HBV transmission, particularly vertical transmission and improving maternal and child health outcomes in high‐burden regions like Nigeria.

## Author Contributions

Elijah Kolawole Oladipo contributed to the study's conception and supervision. Material preparation and data collection were performed by Chinomso Joel Ukagebu, Favour Oluwadara Bamigboye. Analysis was performed by Joel Chimezie Ukaegbu and Jude Oluwapelumi Alao. The first draft of the manuscript was written by Jude Oluwapelumi Alao. All authors commented on previous versions of the manuscript. All authors read and approved the final manuscript.

## Conflicts of Interest

The authors declare no conflicts of interest.

## Data Availability

Data sharing is not applicable to this article as no new data were created or analyzed in this study.
